# Patients with chronic ankle instability exhibit increased sensorimotor cortex, dorsolateral prefrontal cortex, and superior temporal gyrus activation during single-leg stance: a functional near-infrared spectroscopy study

**DOI:** 10.3389/fnhum.2025.1705212

**Published:** 2026-01-26

**Authors:** Huimin Xie, Xuejiao Li, Zihan Wang, Yan Gao, Lining Zhang

**Affiliations:** 1 Department of Rehabilitation, the First Medical Center, Chinese PLA General Hospital, Beijing, China; 2 Office of National Center for Neurological Disorders, Beijing Tiantan Hospital, Capital Medical University, Beijing, China; 3 Hebei University of Chinese Medicine Fourth Hospital, Shijiazhuang, China; 4 First Hospital of Qinhuangdao, Qinhuangdao, China

**Keywords:** balance, chronic ankle instability, dorsolateral prefrontal cortex, sensorimotor cortex, single-leg stance, superior temporal gyrus, functional near infrared spectroscopy

## Abstract

**Introduction:**

Balance deficits are associated with clinical manifestations in patients with chronic ankle instability (CAI); however, evidence of cortical plasticity related to balance control remains insufficient. This study aimed to evaluate cortical activity and balance differences between patients with CAI and healthy individuals during single-leg stance, with or without vision, and to explore the correlations between brain blood flow signals and homeostatic function to elucidate the neurophysiological changes in balance control.

**Methods:**

This cohort study involved 30 patients with CAI from a single hospital and 30 age-matched healthy controls. Cortical activity in the sensorimotor cortex (SMC), dorsolateral prefrontal cortex (DLPFC), and superior temporal gyrus (STG) was measured using functional near-infrared spectroscopy. The activity ranges of the center of mass (COM), acceleration, and acceleration extremes were calculated using wearable inertial sensors. Moreover, scale assessments (visual analog scale, foot and ankle ability measure, and Cumberland Ankle Instability Tool) and functional tests (classical balance, Y-balance, multi-directional reach tests, and timed up-and-go tests) were conducted.

**Results:**

Significant between-group effects were observed for the root mean square (RMS) centroid acceleration in the anteroposterior (AP) direction (*F* = 5.51, *p* = 0.02), whereas within- and between-group differences existed for RMS centroid acceleration in the mediolateral (ML) direction (*F* = 3.56, *p* = 0.03; *F* = 8.5, *p* = 0.004). Significant within- and between-group differences were identified for peak acceleration magnitude (Acc_max) –AP (*F* = 7.85, *p* = 0.001; *F* = 11.83, *p* = 0.001) and Acc_max–ML (*F* = 15.64, *p* = 0.0001; *F* = 5.06, *p* = 0.026). The oxyhemoglobin concentration change (ΔHbO_2_) was significantly greater in patients with CAI than in healthy controls during single-leg stance; between-group differences were identified in the STG–right cerebral hemisphere (R) (*F* = 10.25, *p* = 0.002), DLPFC–R (*F* = 50.99, *p* = 0.001), SMC–R (*F* = 27.48, *p* = 0.0001), STG–left cerebral hemisphere (L) (*F* = 13.6, *p* = 0.0001), DLPFC–L (*F* = 24.21, *p* = 0.0001), and SMC–L (*F* = 29.75, *p* = 0.0001); within-group differences in the SMC–L (*F* = 9.92, *p* = 0.0001); and interaction effects in the STG–R (*F* = 5.73, *p* = 0.004). During right-leg stance with the eyes closed, RMS–ML and Acc_max–ML exhibited a high positive correlation with ΔHbO_2_ in the STG–L (RMS–ML: *p* = 0.001, *r* = 0.72; Acc_max–ML: *p* = 0.001, *r* = 0.74).

**Conclusion:**

Patients with CAI exhibited lower balance ability and greater COM bias than healthy controls, with increased bilateral activation in brain regions, regardless of which limb was elevated; the results were more pronounced with vision inhibited. Enhanced brain activity was positively correlated with COM changes. Functional near-infrared spectroscopy and wearable inertial sensors can detect balance in patients with CAI.

## 1 Introduction

Ankle sprains are among the most common sports injuries in daily life, military training, and competitive sports, with an incidence of approximately 2.15 per 1,000 person-years (Herzog et al., 2019; Drakos et al., 2022). Up to 70% of patients with acute lateral ankle sprains may develop chronic ankle instability (CAI) shortly after the initial injury (Altomare et al., 2022). Evidence suggests that dyskinesia following ankle injury may lead to adaptive changes in the central nervous system, particularly adaptive remodeling of the cerebral cortex (Kim et al., 2019; Nanbancha et al., 2019). It has been suggested that balance control deficits in patients with CAI may be related to functional and structural changes in the sensorimotor processing brain regions (Maricot et al., 2023).

The single-leg stance (SLS) balance performance test is a good indicator of balance ability (Ema et al., 2016); thus, the classic SLS was implemented as the task design in the current study. Herold et al. (2017) found that direct motor networks [e.g., primary motor cortex (M1) and cerebellum] as well as indirect motor networks, including the prefrontal cortex (PFC) and supplementary motor area, are responsible for balance control. Moreover, vestibular brain regions—the superior temporal gyrus and supramarginal gyrus—play significant roles in balance tasks, especially when visual inputs are diminished (Beer et al., 2023). Therefore, we believe that examining the activation level of these brain regions in patients with CAI during balance tasks effectively reflects the response level of relevant functional brain areas to balance as well as the extent of balance ability impairment.

Functional near-infrared spectroscopy (fNIRS) is a well-established neuroimaging modality for real-time monitoring of brain activity, with relatively high spatial sampling and temporal resolution (Sakai, 2022; Eastmond et al., 2022). Its portability, timeliness, and exercise tolerance make fNIRS an optimal tool for studying balance, posture, and gait (Perrey, 2008; Herold et al., 2017; Menant et al., 2020). fNIRS has been widely used to explore the relationship between diseases and balance ability, and to monitor improvements in balance ability after treatment, such as in Parkinson’s disease, adolescent idiopathic scoliosis, stroke, and hypertension (Kohli et al., 2025; Suzen et al., 2025; Fan et al., 2024; Stuart et al., 2018). Wearable inertial sensors are commonly used to detect balance and estimate proprioceptive abilities, making them widely used in balance tasks (Petraglia et al., 2019). Specifically, these sensors can be used to assess kinematic parameters during balance tasks in people with CAI (Deodato et al., 2023).

However, there is currently a lack of research on the relationship between real-time balance and neurophysiological changes after CAI. Therefore, this study aimed to explore the neurophysiological mechanisms underlying CAI, enhance the current understanding of CAI-induced neural remodeling, and establish a theoretical foundation for future neural rehabilitation. To investigate the relationship between balance ability and neural remodeling, we employed wearable inertial sensors and fNIRS for synchronous monitoring and conducted a data coupling analysis.

## 2 Materials and methods

### 2.1 Participants

Thirty patients diagnosed with right-sided CAI at Chinese PLA General Hospital between September 2021 and December 2023 were enrolled. To avoid excessive grouping and scenario discussions, and since most people are right-side dominant, only patients with right-sided CAI were included. The dominant leg was defined as the leg that the patient habitually used to kick a ball (Li et al., 2018). A control group comprising 30 healthy, age-matched participants was formed. The inclusion criteria for patients were as follows: (1) the first sprain occurred ≥ 12 months prior to the scan; (2) at least one acute ankle inversion sprain resulting in swelling, pain, and dysfunction had occurred; (3) the most recent injury occurred > 2 months prior to the scan; (4) at least two episodes of ankle “give way” had occurred in the past 6 months; (5) a score of < 24 on the Cumberland Ankle Instability Tool (CAIT); 6) a score of < 90% on the Foot and Ankle Ability Measure (FAAM) –activities of daily living (ADL) and—exercise scales, and < 80% on the FAAM–SPORTS scale; (7) no other chronic medical conditions and no intracranial or psychological disorders; and (8) the affected limb was identified as the right lower extremity (Gribble et al., 2014). The exclusion criteria were as follows: (1) acute injury to a musculoskeletal structure of a lower extremity joint other than the ankle within 3 months prior to testing and (2) presence of other chronic medical conditions or intracranial or psychological disorders. The experimental study was approved by the Ethics Committee of Chinese PLA General Hospital (S2022-102-02) and adhered to the tenets of the Declaration of Helsinki. All participants provided informed consent.

### 2.2 Scale assessments and functional testing

Participants’ clinical data, including body mass index (BMI), age, disease duration, diagnosis, and imaging findings, were collected. The scores of the scale assessments, including the visual analog scale, FAAM–ADL, FAAM–SPORTS, and CAIT, were recorded. Functional tests included classical balance tests, the Y-balance test, multi-directional reach test (MDRT), and the timed up-and-go (TUG) test. The Y-balance test results included the anterior reach difference (Y–A), posterior lateral difference (Y–PL), posterior medial difference (Y–PM), and total score. The MDRT assessed reach in four directions: forward reach, backward reach (BR), leftward reach (LR), and rightward reach (FR).

### 2.3 Data acquisition

#### 2.3.1 Inertial sensor-based assessment

Six wireless inertial measurement unit (IMU) sensors (Type DE-A, Suzhou Hengpin Medical Technology Co., Ltd., China) were used to acquire kinematic signals during the SLS test. The sensors were placed on the chest, waist, bilateral upper arms, and thighs, and were connected to a sensory balance testing system. Each sensor unit contained a three-dimensional gyroscope, accelerometer, and magnetometer. The activity ranges of the center of mass (COM), acceleration, and acceleration extremes, and of other data were calculated using computer software with a sampling frequency of 100 Hz. The data were transmitted to the computer operating platform in real time via Bluetooth and record it.

#### 2.3.2 Functional near-infrared spectroscopy assessment

**FIGURE 1:**
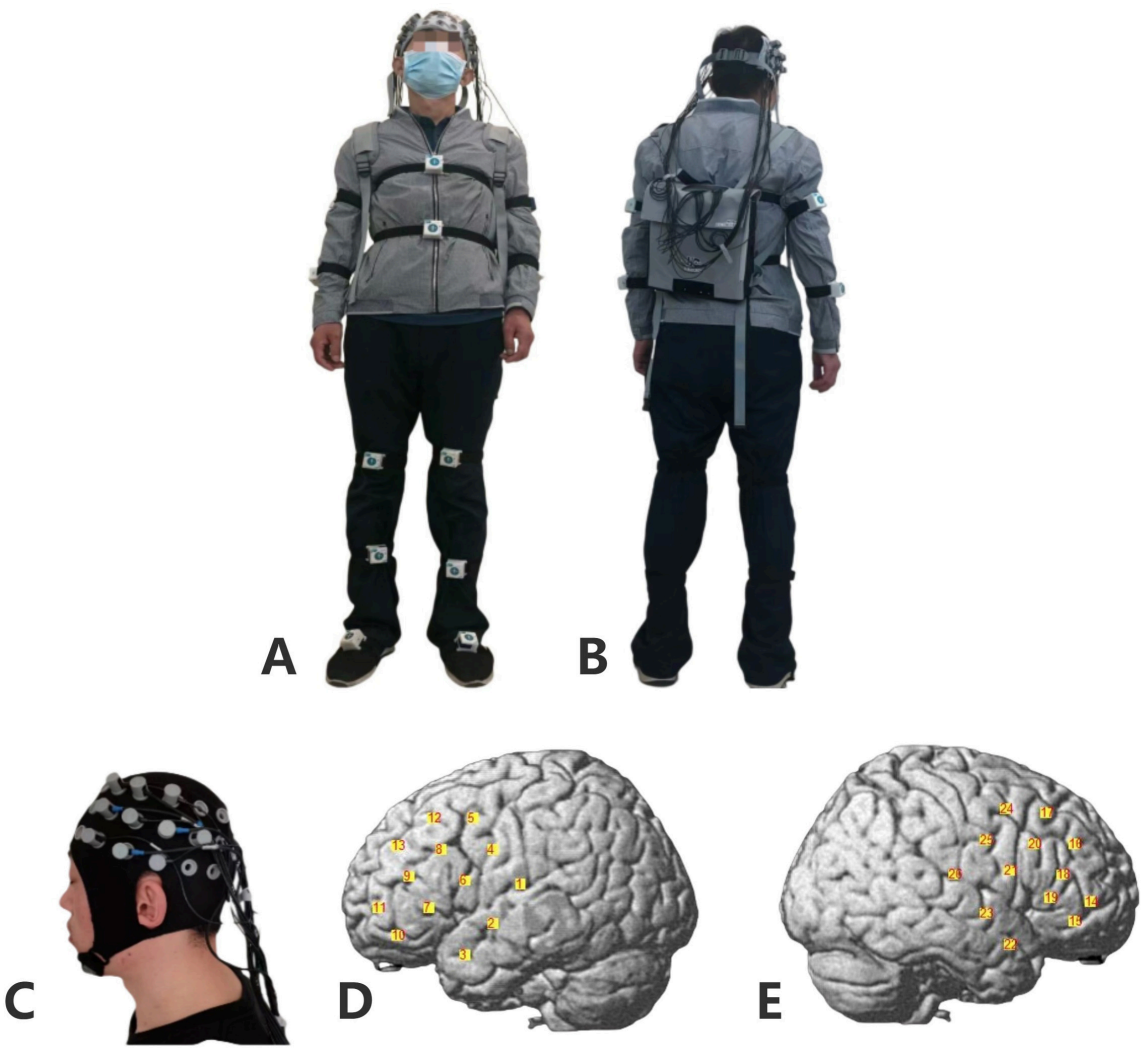
fNIRS positioning. **(A)** Real view during testing (front view). **(B)** Real view during testing (back view). **(C)** Near-infrared equipment cap. **(D)** Schematic diagram of channel positions in the left cerebral hemisphere. **(E)** Schematic diagram of channel positions in the right cerebral hemisphere. fNIRS, functional near-infrared spectroscopy.

A portable multichannel near-infrared functional brain imaging device (NirSmart; Danyang Huichuang Medical Equipment Co., Ltd., China) was used to record the brain activity. Following the 10/20 international system, 20 probes (eight sources and 12 detectors) were placed to cover the partial frontal, temporal, and parietal lobes, resulting in 26 channels with simultaneous frequency-encoded dual-wavelength illumination at 760 and 850 nm. The between-probe distance was set to 30 mm, and the entire channel sampling rate of the equipment exceeded 11 Hz (Li et al., 2020). To avoid errors from the light source or receiver movement, a whole-brain custom snap-on cap was used, and the patient carried the portable device on their back during the procedure. To examine the hemodynamic response, the change in the oxygenated hemoglobin level (ΔHbO_2_) was recorded. The mean value of HbO within the last 5 s of each rest time was selected as a baseline for correction. An international 10–20 electroencephalogram (EEG) acquisition system (NirSmart; Danyang Huichuang Medical Equipment Co., Ltd., China) was used for electronic positioning, and the standard cap was customized the cap in a standardized manner (Tzourio-Mazoyer et al., 2002). Six regions of interest were categorized: Left/right dorsolateral prefrontal lobes (DLPFC; Channel 11–13, Channel 14, 16,17), left/right superior temporal gyrus (STG; Channel 1–3, Channel 22, 23, 26), and left/right partial motor sensory areas (SMC; Channel 4–6, Channel 21, 24, 25) (Figure 1).

### 2.4 Experimental procedure

**FIGURE 2:**
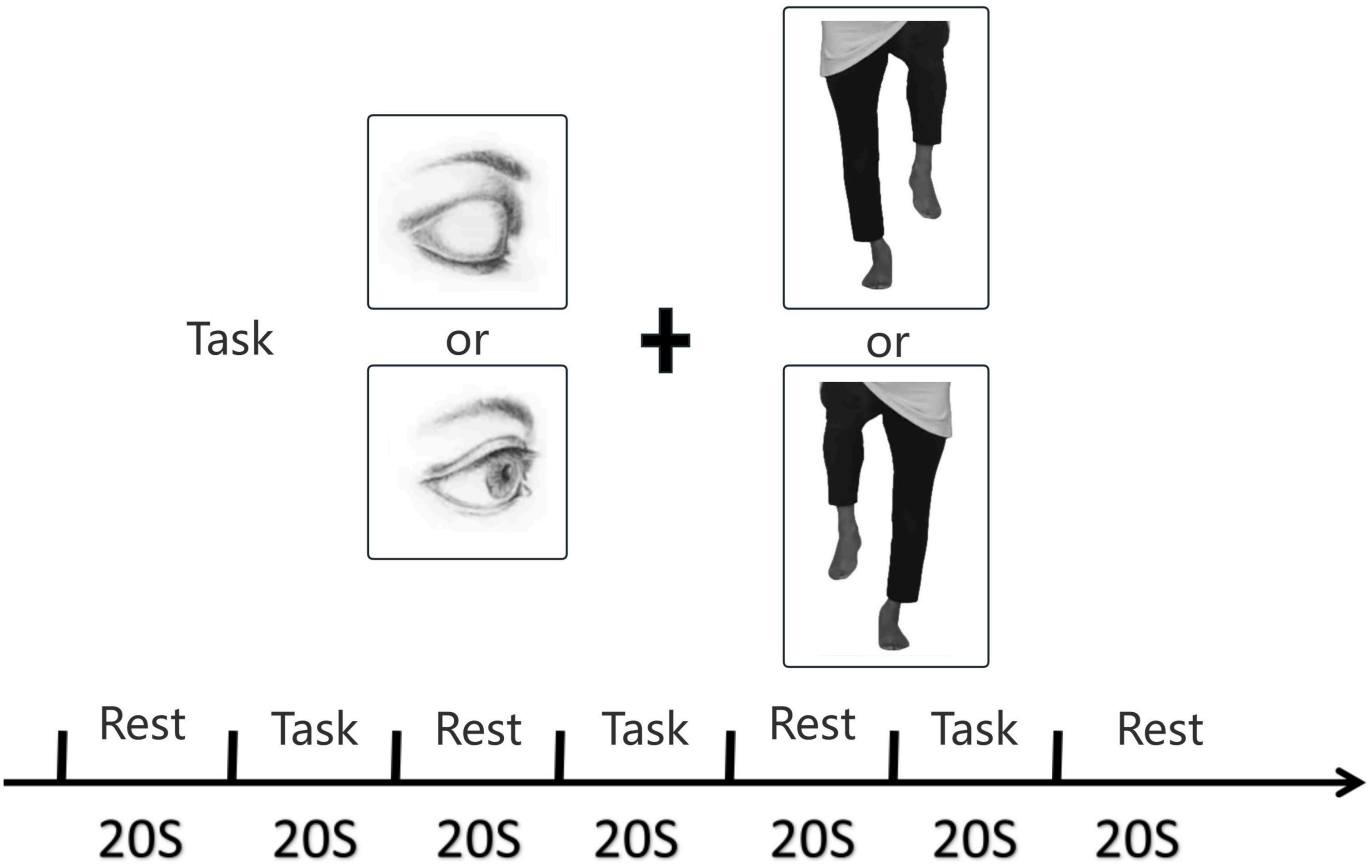
Experimental setup.

The SLS test was used to assess differences in blood flow across functional brain regions during static balance (Omaña et al., 2021). Considering the effects of vision and different leg supports, the test was refined into four single-leg support paradigms: (1) right lower extremity as the stance limb, left limb elevated, eyes open (RO); (2) right lower extremity as the stance limb, left limb elevated, eyes closed (RC); (3) left lower extremity as the stance limb, right limb elevated, eyes open (LO); and (4) left lower extremity as the stance limb, right limb elevated, eyes closed (LC). The healthy control group used their dominant leg as the stance limb, with their eyes either open or closed. Each task was repeated three times. Before the experiment, the participants were instructed on how to stand on one foot; for example, to keep the upper body centered and slowly lift one foot forward approximately 10 cm from the ground to maintain balance. The environment was quiet and moderately lit during the experiment. Each trial lasted 140 s per condition; the first 20 s was the baseline period, followed by 20 s of single-leg standing and then 20 s of rest. The experimental setup is shown in Figure 2. During the baseline and rest periods, all participants stood upright in a natural two-foot stance. The synchronization between IMU and fNIRS signals is accomplished through manual marking by the operator.

### 2.5 Data processing

#### 2.5.1 Hemodynamics

MATLAB 2020a and HOMER2 software was used for fNIRS data processing. Bad channels and artifacts in the fNIRS data were manually identified and labeled. A channel was labeled as bad if its mean light intensity was < 1 or > 1,000, or if its CV% exceeded 10%, using the enPruneChannel function (Piper et al., 2014). Raw optical intensity data were converted into optical density for subsequent calculations. We removed the effects caused by physiological factors such as heartbeat and breathing through artifact correction and filtering (Yücel et al., 2021). Using the hmrMotionArtifactByChannel function, each channel was examined, and any data values showing fluctuations exceeding five times the mean or more than 50 times the standard deviation in 2 s were marked as artifacts, with 4 s around that point flagged accordingly. The hmrBandpassFilt function was used to remove some noisesignals, including high-frequency (≥0.2 Hz; heartbeat, respiratory rate, and instrument noise) and very low frequency (<0.01 Hz; yascular and metabolic oscillations) (Hocke et al., 2018). Noise-reduced optical density values were converted into HbO_2_ using the Modified Beer–Lambert equation. The differential path length factor was set to 6.0. HbO2 was chosen as a metric because HbO2 signals have a better signal-to-noise ratio (Kumar et al., 2017), higher reproducibility (Zhang et al., 2011), are more sensitive indicators of local blood flow changes (Hoshi, 2003), and have demonstrated superiority in assessing functional activity (Liu et al., 2024). The mean HbO2 concentrations during the period from 5 s after each task started to 10 s after each task ended were measured (Piper et al., 2014). Map the calculated indicator values of the channels onto the standard brain according to the MNI coordinates of the channels, and then use BrainNet toolbox in MATLAB to create a visual rendering.

#### 2.5.2 Center of mass

Sensor data were processed into digital information, and a formula was used to calculate the difference in body sway, which represented the balance differences. The root mean square (RMS) centroid acceleration was computed from the raw sensor data. The two axes represent left–right and front–back. Over 20 s, the sensor recorded 400 COM acceleration data points in each direction. The formulae used to calculate RMS–AP and RMS–ML were as follows:


$$\mathrm{RMS}-\mathrm{AP}=\sqrt{\frac{\sum {\left(x_{i}-x\right)}^{2}}{N-1;}}\,\mathrm{RMS}-\mathrm{ML}=\sqrt{\frac{\sum {\left(y_{i}-y\right)}^{2}}{N-1}.}$$

The peak acceleration magnitude (Acc_max) was defined as the absolute value of the maximum or minimum centroid acceleration. Data in the anteroposterior (AP) and mediolateral (ML) directions were analyzed.

### 2.6 Statistical analyses

Data analysis was performed using SPSS 26 (IMB Corp., Armony, NY), and GraphPad Prism 8 (GraphPad Software Corp., Boston, MA) was used for plotting. Continuous variables are expressed as mean ± standard deviation and were compared between the two groups using independent samples *t*-tests. Categorical variables were analyzed using the chi-squared test. Levene’s test for equality of variances was applied. Kolmogorov-Smirnov Test is used to check for normal distribution. Mann-Whitney U statistics were calculated for results if the data did not follow a normal distribution. Differences between three groups were evaluated by analysis of variance (ANOVA). Cohen’s d was used for effect size detection in *t*-tests, while partial eta 2 was used in ANOVA. Pearson’s correlation coefficient (*r*) was used to explore the relationships between variables. A *p*-value < 0.05 indicated statistical significance.

## 3 Results

### 3.1 Demographic data

Thirty-two patients met the inclusion criteria, of whom two could not tolerate wearing the device and withdrew from the experiment. Ultimately, 30 male patients with right-sided CAI (age 33.5 ± 10.4 years) and 30 male, age-matched, healthy controls (HCs) (age 30.33 ± 6.16 years) were included (*p* = 0.16, Cohen’s *d* = 0.37). The study duration was 36.96 ± 45.78 months. The BMI of CAI group was 23.06 ± 2.02 kg/m^2^, while that of the HC group was 21.07 ± 1.91 kg/m^2^ (*p* = 0.001, Cohen’s *d* = 1.01). The CAIT, FAAM-ADL, and FAAM-SPORTS scores in the CAI group were 13.23 ± 6.08, 61.43 ± 15.84, and 15.38 ± 8.18, respectively. The Y-balance test and MDRT scores are presented in Table 1.

**TABLE 1:** The demographic, scale assessment, and functional testing data of the participants.

	CAI group	HC group	*t*	*p*	Cohen’s d	95% CI
Age (years)	33.5 ± 10.4	30.33 ± 6.16	–1.44	0.16	0.37	–7.58, 1.24
BMI (kg/m^2^)	23.06 ± 2.02	21.07 ± 1.91	3.90	0.001**	1.01	0.96, 2.99
Duration (months)	36.96 ± 45.78	N/A	N/A	N/A	N/A	N/A
CAIT	13.23 ± 6.08	N/A	N/A	N/A	N/A	N/A
FAAM–ADL	61.43 ± 15.84	N/A	N/A	N/A	N/A	N/A
FAAM–SPORTS	15.38 ± 8.18	N/A	N/A	N/A	N/A	N/A
Y-Balance	83.02 ± 15.60	101.02 ± 9.15	–5.45	0.001**	1.4	−24.62, −11.40
Y–A (cm)	7.58 ± 5.51	3.94 ± 5.14	2.21	0.031*	0.68	0.34, 6.93
Y–PL (cm)	8.43 ± 6.67	3.23 ± 2.74	3.95	0.001**	1.02	2.56, 7.84
Y–PM (cm)	9.69 ± 8.79	3.07 ± 3.27	3.86	0.001**	1.00	3.20, 10.06
TUG (s)	8.85 ± 0.79	6.60 ± 0.62	12.25	0.001**	1.72	1.88, 2.62
**MDRT (cm)**						
FR	40.15 ± 6.37	37.34 ± 4.82	1.92	0.06	0.50	−0.12, 5.74
BR	21.82 ± 5.34	25.04 ± 5.64	–2.27	0.027*	0.59	−6.06, −0.38
RR	24.16 ± 4.60	26.42 ± 4.98	–1.83	0.072	0.47	−4.74, 0.21
LR	25.16 ± 3.52	25.73 ± 4.58	–0.54	0.59	0.14	−2.68, 1.54

ADL, Activities of daily living; BMI, Body mass index; CAIT, Cumberland Ankle Instability Tool; CAI, Chronic ankle instability; CI, Confidence interval; FAAM, Foot and Ankle Ability Measure; HC, Healthy controls; N/A, Not applicable; TUG, Timed Up-and-Go test; MDRT, Multi-directional reach test; BR, Backward reach; LR, Leftward reach; FR, Forward reach; RR, Rightward reach; Y−A, Y-Balance test anterior reach difference value; Y−PL, Y-Balance test posterolateral reach difference value; Y−PM, Y-Balance test posteromedial reach difference value; *p ≤ 0.05, **p ≤ 0.01.

### 3.2 Inertial measurement unit data

Patients with CAI exhibited poorer balance compared with HCs, regardless of whether they were supported by an affected or healthy leg. Significant between-group effects were observed for RMS–AP (*F* = 5.51, *p* = 0.02), whereas within- and between-group differences were observed for RMS–ML (*F* = 3.56, *p* = 0.03; *F* = 8.5, *p* = 0.004). Additionally, significant within- and between-group differences were identified for Acc_max–AP (*F* = 7.85, *p* = 0.001; *F* = 11.83, *p* = 0.001) and Acc_max–ML (*F* = 15.64, *p* = 0.0001; *F* = 5.06, *p* = 0.026) (Table 2).

**TABLE 2:** Inertial measurement unit data for patients and healthy controls.

Group	RMS−AP (m/s^2^)	RMS−ML (m/s^2^)	Acc_max−AP (m/s^2^)	Acc_max−ML (m/s^2^)
**Open eyes**				
LO	0.10 ± 0.05	0.12 ± 0.08	0.5 ± 0.53	0.38 ± 0.37
RO	0.14 ± 0.16	0.14 ± 0.07	0.68 ± 0.41	0.7 ± 0.58
DO	0.09 ± 0.06	0.09 ± 0.05	0.31 ± 0.2	0.19 ± 0.1
**Closed eyes**				
LC	0.14 ± 0.02	0.13 ± 0.1	0.71 ± 0.52	0.58 ± 0.32
RC	0.15 ± 0.02	0.17 ± 0.1	0.81 ± 0.42	0.71 ± 0.63
DC	0.17 ± 0.23	0.15 ± 0.05	0.58 ± 0.22	0.58 ± 0.44
**ANOVA**				
Within group variance (F, *p,η^2^_*Partial*_*)	0.64, 0.53, 0.007	3.56, 0.03*, 0.04	7.85, 0.001**, 0.083	15.64, 0.001**, 0.152
Between group variance (F, *p,η^2^_*Partial*_*)	5.51, 0.02*, 0.31	8.5, 0.004**, 0.47	11.83, 0.001**, 0.064	5.06, 0.026**, 0.28
Interaction effect (F, *p,η^2^_*Partial*_*)	1.44, 0.24, 0.016	1.21, 0.3, 0.014	0.61, 0.55, 0.07	1.12, 0.33, 0.013

AP, Anteroposterior; ML, Mediolateral; RO, Right lower extremity is the stance limb, while the left limb is the elevated limb with the eyes open; RC, Right lower extremity is the stance limb, while the left limb is the elevated limb with the eyes closed; LO, Left lower extremity is the stance limb, while the right limb is the elevated limb with the eyes open; LC, Left lower extremity is the stance limb, while the right limb is the elevated limb with the eyes closed; DO, Healthy participants used the dominant leg as a stance limb with the eyes open; DC, Healthy participants used the dominant leg as a stance limb with the eyes open; RMS, Root mean square of centroid acceleration; Acc_max, Centroid acceleration extreme. *p ≤ 0.05, **p ≤ 0.01.

### 3.3 Hemodynamic data

**FIGURE 3:**
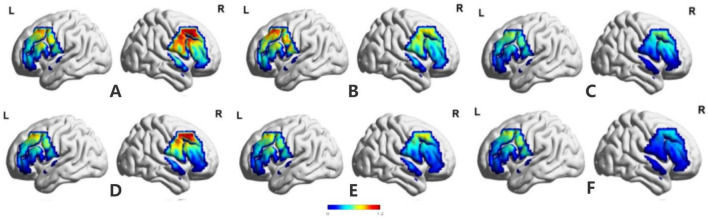
Brain activation maps of patients with CAI and healthy participants during single-leg stance. **(A)** Patients with CAI used the left lower extremity as the stance limb when the eyes were closed (LC). **(B)** Patients with CAI used the right lower extremity as the stance limb when the eyes were closed (RC). **(C)** Healthy participants used the dominant leg as the stance limb when the eyes were closed. **(D)** Patients with CAI used the left lower extremity as the stance limb when the eyes were open (LO). **(E)** Patients with CAI used the right lower extremity as the stance limb when the eyes were open (RO). **(F)** Healthy participants used the dominant leg as the stance limb when the eyes were open. ΔHbO_2_, oxyhemoglobin concentration change; CAI, chronic ankle instability.

Significant between-group differences in the ΔHbO_2_ were found in the STG–R (*F* = 10.25, *p* = 0.002), DLPFC–R (*F* = 50.99, *p* = 0.001), SMC–R (*F* = 27.48, *p* = 0.0001), STG–L (*F* = 13.6, *p* = 0.0001), DLPFC–L (*F* = 24.21, *p* = 0.0001), and SMC–L (*F* = 29.75, *p* = 0.0001); within-group differences were found in the SMC–L (*F* = 9.92, *p* = 0.0001); and an interaction effect was found in the STG–R (*F* = 5.73, *p* = 0.004). The CAI group exhibited a higher ΔHbO_2_ than the HC group (Table 3 and Figure 3).

**TABLE 3:** Hemodynamics data for patients and healthy controls.

Δ[HbO_2_] (μmoL)	STG−R	DLPFC−R	SMC−R	STG−L	DLPFC−L	SMC−L
**Open eyes**						
RO	0.16 ± 0.42	0.22 ± 0.38	0.46 ± 0.48	0.05 ± 0.51	0.18 ± 0.38	0.53 ± 0.46
LO	0.28 ± 0.29	0.38 ± 0.4	0.59 ± 0.54	0.27 ± 0.35	0.33 ± 0.37	0.77 ± 0.61
DO	0.29 ± 0.46	0.2 ± 0.26	0.38 ± 0.28	0.11 ± 0.27	0.18 ± 0.32	0.38 ± 0.45
**Closed eyes**						
RC	0.59 ± 0.37	0.63 ± 0.48	0.91 ± 0.45	0.48 ± 0.41	0.60 ± 0.39	0.95 ± 0.48
LC	0.42 ± 0.28	0.58 ± 0.42	0.85 ± 0.49	0.41 ± 0.39	0.59 ± 0.46	1.1 ± 0.56
DC	0.26 ± 0.37	0.43 ± 0.32	0.75 ± 0.46	0.21 ± 0.47	0.36 ± 0.38	0.67 ± 0.39
**ANOVA**						
Within group variance (F, *p,η^2^_*Partial*_*)	1.21, 0.3, 0.014	2.79, 0.064, 0.031	1.88, 0.155, 0.021	2.74, 0.067, 0.031	3.81, 0.024*, 0.042	9.92, 0.001**, 0.102
Between group variance (F, *p,η^2^_*Partial*_*)	10.25, 0.002**, 0.056	50.99, 0.001**, 0.12	27.48, 0.001**, 0.136	13.60, 0.001**, 0.072	24.21, 0.001**, 0.122	29.75, 0.001**, 0.107
Interaction effect (F, *p,η^2^_*Partial*_*)	5.73, 0.004**, 0.062	0.71, 0.583, 0.017	0.66, 0.517, 0.008	2.62, 0.076, 0.029	1.38, 0.255, 0.016	0.28, 0.755, 0.003

TP−L, Left temporal-parietal areas; DLPFC−L, Left granular dorsolateral prefrontal cortex; SMC−L, Left sensorimotor cortex; TP−R, Right temporal-parietal areas; DLPFC−R, Right granular dorsolateral prefrontal cortex; SMC−R, Right sensorimotor cortex; RO, Right lower extremity is the stance limb, while the left limb is the elevated limb with the eyes open; RC, Right lower extremity is the stance limb, while the left limb is the elevated limb with the eyes closed; LO, Left lower extremity is the stance limb, while the right limb is the elevated limb with the eyes open; LC, Left lower extremity is the stance limb, while the right limb is the elevated limb with the eyes closed; DO, Healthy participants used the dominant leg as a stance limb with the eyes open; DC, Healthy participants used the dominant leg as a stance limb with the eyes open. *p ≤ 0.05, **p ≤ 0.01.

### 3.4 Correlations between hemodynamic and IMU data

The correlations between hemodynamic and IMU data for each of the four SLS test conditions were as follows:

LO: RMS–ML showed a moderate positive correlation with ΔHbO_2_ in the SMC–R (*p* = 0.005, *r* = 0.49).

RO: No significant correlations were observed.

LC: RMS–ML exhibited a moderate positive correlation with ΔHbO_2_ in the STG–R (*p* = 0.02, *r* = 0.42), DLPFC–R (*p* = 0.001, *r* = 0.63), STG–L (*p* = 0.004, *r* = 0.5), and DLPFC–L (*p* = 0.003, *r* = 0.53). Acc_max–AP exhibited a moderate positive correlation with ΔHbO_2_ in the DLPFC–R (*p* = 0.042, *r* = 0.45).

**FIGURE 4:**
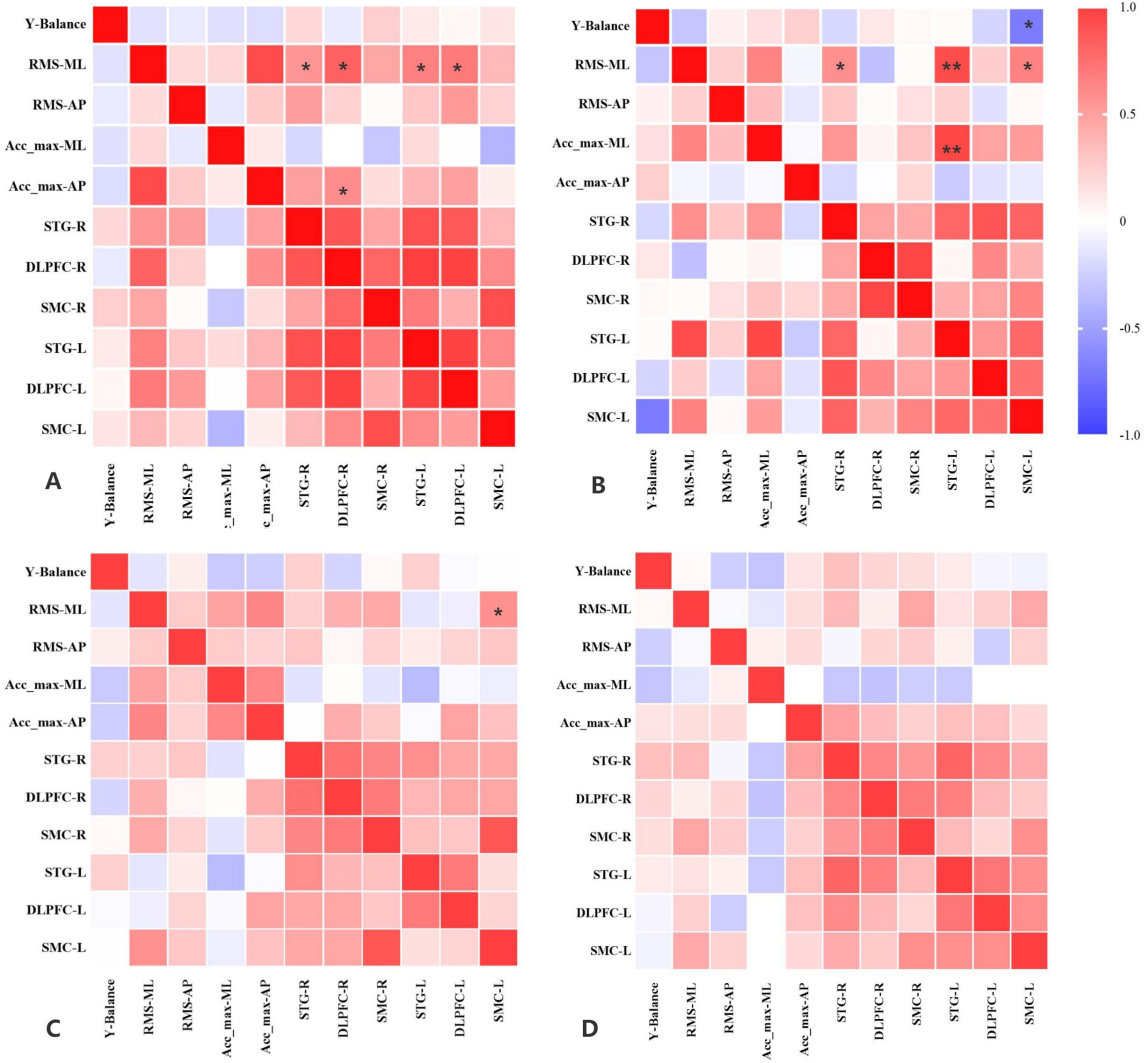
Correlations between hemodynamic and IMU data. **(A)** Patients with CAI used the left lower extremity as the stance limb when the eyes were closed (LC). **(B)** Patients with CAI used the right lower extremity as the stance limb when the eyes were closed (RC). **(C)** Patients with CAI used the right lower extremity as the stance limb when the eyes were open (RO). **(D)** Patients with CAI used the left lower extremity as the stance limb when the eyes were open (LO). CAI, chronic ankle instability. *Moderate correlation; **high correlation.

RC: Y-balance test scores showed a moderate negative correlation with ΔHbO_2_ in the STG–L (*p* = 0.003, *r* = −0.52). RMS–ML had a moderate positive correlation with ΔHbO_2_ in the STG–R (*p* = 0.015, *r* = 0.44) and SMC–L (*p* = 0.005, *r* = 0.49). RMS–ML exhibited a high positive correlation with ΔHbO_2_ in the STG–L (*p* = 0.001, *r* = 0.72). Acc_max–ML demonstrated a high positive correlation with the HbO_2_ level in the STG–L (*p* = 0.001, *r* = 0.74) (Figure 4).

## 4 Discussion

In this study, 30 patients with right-sided CAI and 30 age-matched HCs were recruited, effectively controlling for age-related confounding variables. Bilateral cerebral hemodynamic profiling was performed along with standardized SLS protocols to assess both affected and contralateral limbs. This approach overcame the methodological limitations of prior unilateral assessment paradigms in both neurological and biomechanical domains. The CAI group showed significantly diminished composite scores on the Y-balance test, with directional deficits persisting across all three reach vectors, compared with those of the HC group. Additionally, significant inter-group disparities were identified in the TUG and MDRT–BR trials. The Cohen’s d values for these differences range from 0.59 to 1.72, indicating a high likelihood that the differences have a true statistical effect. These multidirectional deficits across assessment paradigms substantiate the impaired postural control observed in patients with CAI, indicating underlying sensorimotor integration deficiencies.

Contemporary biomechanical assessments have increasingly used IMU, allowing for the precise quantification of COM oscillations and triaxial angular velocity dynamics during balance-challenging tasks, instead of simply relying on measures of swing amplitude (Lin et al., 2024). Key biomechanical parameters include RMS acceleration, instantaneous acceleration variation rate, Acc_max, mean displacement trajectory, mean resultant velocity, and spectral dominant frequency of COM sway (Ghattas and Jarvis, 2024). Onuma et al. (2023) analyzed the peak magnitude of lumbar acceleration in the side-stance direction as an anticipatory postural adjustment feature and suggested the simplicity and suitability of this method for continuous patient monitoring. Mohebbi et al. (2022) conducted a randomized crossover study employing virtual reality-induced visual perturbations to quantify postural responses. Their findings revealed a positive linear correlation between the visual stimulus amplitude and hip displacement RMS values. These authors specifically quantified neuromuscular responses through RMS and Acc_max values; similarly, in our present study, we assessed these two biomechanical indicators. Quantitative analysis revealed significantly elevated postural instability metrics in patients with CAI compared with those in HCs, regardless of weight-bearing limb status, consistent with previous research (Deodato et al., 2023). This phenomenon may account for the high incidence of subsequent bilateral ankle sprains in populations with unilateral CAI. Both patients with CAI and HCs exhibited greater COM displacement when standing on one-leg with their eyes closed compared to with their eyes open. This is because the removal of visual assistance reduces balance stability (Tomomitsu et al., 2013; Onofrei and Amaricai, 2022). Moreover, patients with CAI exhibited greater RMS–ML, Acc_max–AP, and Acc_max–ML values than HCs when their eyes were closed. In the between-group comparisons and within-group comparisons of Acc_max−AP and Acc_max−ML, the partial eta squared values were all > 0.06, indicating that the differences between the groups had a moderate to high effect. This suggests that patients with CAI may rely more heavily on visual aids to maintain their balance. Onofrei and Amaricai (2022) studied posture balance in healthy individuals and found a large lateral deviation in the mean center of pressure, consistent with our results. These findings indicate that rehabilitation strategies for patients with CAI should not only focus on balance movements with vision but also, under the premise of safety, should incorporate movements without visual assistance as much as possible.

In the present study, bilateral cerebral oxygenation increased during one-legged stance. The effect indicators also show that the correlation between the variables in the groups is moderate to high. Previously, Chen et al. (2020) summarized cortical activation differences—including in the bilateral frontal areas, SMG, STG, supplementary motor area, DLPFC, and inferior frontal gyrus—during balance tasks in healthy individuals. Owing to limited equipment availability, the DLPFC, STG, and SMC brain regions were chosen in the current study, based on pre-experimental findings. We observed that the patients with CAI exhibited higher activation in these regions compared with the HCs. Moreover, the patients with CAI exhibited increased cerebral oxygenation in the DLPFC, STG, and SMC brain regions, regardless of whether they were supported by the affected or healthy leg. This suggests increased activation of cognitive, vestibular, and motor sensory-related brain regions in patients with CAI during balance tasks, indicating their reliance on an altered cortical activation strategy to maintain homeostasis. In a similar study conducted by Liu et al. (2024), patients with CAI exhibited more sensorimotor cortex activity—including in the pre-motor cortex, supplementary motor area, primary motor cortex, and primary somatosensory cortex—during one-legged stance. Ma et al. (2023) also found that patients with ankle injury may experience increased activation of the primary somatosensory cortex and superior temporal gyrus, consistent with the findings of the present study. Our findings, combined with those of previous studies, suggest that the increased activation of these functional brain regions in patients with CAI may reflect a compensatory mechanism that provides functional benefits (Xie et al., 2022; Ma et al., 2023). In the future, rehabilitation strategies could be adjusted to include early mobilization instead of immobilization, earlier initiation of balance training, and non-invasive neuromodulation of relevant functional brain areas.

To maintain posture on a stable support surface, the distribution of sensory information is approximately 70% proprioceptive, 20% vestibular, and 10% visual (Chiba et al., 2016). However, when visual input is absent, proprioception and vestibular sensations are maintained in similar proportions (Riemann and Lephart, 2002). Consequently, in the current study, COM displacement was greater, and blood flow signals in the cortical detection area were enhanced when participants’ eyes were closed compared with when they were open. This suggests that in the absence of visual input, patients with CAI need to activate more vestibular, cognitive, and motor sensory-related brain regions to maintain balance, as supported by previous studies (Lee et al., 2021; Ma et al., 2023; Richer et al., 2024).

To further explore the relationship between balance and brain region activation in patients with CAI, we correlated the patients’ scale, functional test, sensory, and fNIRS data. Accordingly, RMS–ML was positively correlated with ΔHbO_2_ in the bilateral SMC regions when eyes were open, whereas under eyes-closed conditions, RMS–ML showed significant positive correlations with both the bilateral SMC and STG areas. This suggests that balance is primarily maintained by the activation of vestibular-related brain regions in the absence of visual aids. Additionally, motor control of the distal limb was found to be predominantly attributed to the cortex in the contralateral hemisphere (Lehmann et al., 2021). Our study outcomes largely align with these findings, showing greater activation in the relevant brain regions contralateral to the supported leg during the SLS test. However, this also suggests that, despite the task involving a unilateral distal limb, the relevant brain regions in the cerebral cortex are activated bilaterally. Similar findings were reported by Ruddy et al. (2017) and Ziabari et al. (2022a,b), who noted that CAI alters the kinematics and biomechanics of the uninjured contralateral ankle. Therefore, the increased activation of bilateral brain regions may represent a compensatory strategy to maintain the overall body balance. This indicates that damage to one limb may cause bilateral imbalance. Regarding the abnormally activated brain regions identified in the current experiment, future studies should investigate the possibility of using existing non-invasive neuromodulation techniques (such as transcranial magnetic stimulation and transcranial electrical stimulation) for regulation, or combining them with peripheral rehabilitation strategies to shorten the treatment course or achieve a cure.

### 4.1 Study limitations

First, this study was limited by the fNIRS channel, which prevented the simultaneous monitoring of the activation of more functional brain regions involved in balance control, such as the occipital lobe. Second, this was a cross-sectional study, which cannot infer causality between adaptive brain characteristics and CAI development. Third, other confounding factors, such as physical activity level, were not accounted for; future experiments should conduct stratified analyses. Fourth, ΔHbO2 may have a false positive rate. The deoxyhemoglobin concentration (HHb), and total hemoglobin concentration (HbT) data should also be collected simultaneously (Tachtsidis and Scholkmann, 2016). Finally, the small sample size may have induced some bias.

## 5 Conclusion

Patients with CAI demonstrated lower balance ability than healthy individuals. During the one-legged stance task, patients with CAI exhibited greater COM bias than HCs, along with increased bilateral activation of brain regions—including the STG, DLPFC, and SMC—regardless of whether the affected or unaffected limb was elevated. Moreover, these results were more pronounced during the SLS tests performed without vision, and the increased brain activity was largely positively correlated with the COM alterations. Our findings indicate that fNIRS and wearable inertial sensors can be used to detect balance in patients with CAI.

## Data Availability

The raw data supporting the conclusions of this article will be made available by the authors, without undue reservation.

## References

[B1] Altomare D. Fusco G. Bertolino E. Ranieri R. Sconza C. Lipina M. (2022). Evidence-based treatment choices for acute lateral ankle sprain: a comprehensive systematic review. *Eur. Rev. Med. Pharmacol. Sci*. 26 1876–1884. 10.26355/eurrev_202203_28333 35363336

[B2] Beer A. L. Becker M. Frank S. M. Greenlee M. W. (2023). Vestibular and visual brain areas in the medial cortex of the human brain. *J. Neurophysiol*. 129 948–962. 10.1152/jn.00431.2022 36988202

[B3] Chen X. Lu Y. Bao C. Quan H. Ren J. Wu X. (2020). A systematic study of near-infrared functional brain imaging for balance task in healthy subjects. *Chin. J. Pract. Neurol. Dis.* 12 1100–1105. 10.1177/15459683211028548 34171982

[B4] Chiba R. Takakusaki K. Ota J. Yozu A. Haga N. (2016). Human upright posture control models based on multisensory inputs; in fast and slow dynamics. *Neurosci. Res*. 104 96–104. 10.1016/j.neures.2015.12.002 26746115

[B5] Deodato M. Coan L. Buoite Stella A. Ajčević M. Martini M. Di Lenarda L. (2023). Inertial sensors-based assessment to detect hallmarks of chronic ankle instability during single-leg standing: Is the healthy limb “healthy”? *Clin. Biomech*. 107:106036. 10.1016/j.clinbiomech.2023.106036 37406582

[B6] Drakos M. Hansen O. Kukadia S. (2022). Ankle instability. *Foot Ankle Clin*. 27 371–384. 10.1016/j.fcl.2021.11.025 35680294

[B7] Eastmond C. Subedi A. De S. Intes X. (2022). Deep learning in fNIRS: a review. *Neurophotonics* 9:041411. 10.1117/1.NPh.9.4.041411 35874933 PMC9301871

[B8] Ema R. Saito M. Ohki S. Takayama H. Yamada Y. Akagi R. (2016). Association between rapid force production by the plantar flexors and balance performance in elderly men and women. *Age* 38 475–483. 10.1007/s11357-016-9949-3 27581165 PMC5266226

[B9] Fan W. Zeng Q. Zheng P. Wen S. Li G. Fan T. (2024). Brain activation in older adults with hypertension and normotension during standing balance task: an fNIRS study. *Front. Aging Neurosci*. 16:1458494. 10.3389/fnagi.2024.1458494 39381138 PMC11458469

[B10] Ghattas J. Jarvis D. N. (2024). Validity of inertial measurement units for tracking human motion: a systematic review. *Sports Biomech*. 23 1853–1866. 10.1080/14763141.2021.1990383 34698600

[B11] Gribble P. A. Delahunt E. Bleakley C. M. Caulfield B. Docherty C. L. Fong D. T. (2014). Selection criteria for patients with chronic ankle instability in controlled research: a position statement of the International Ankle Consortium. *J. Athl. Train*. 49 121–127. 10.4085/1062-6050-49.1.14 24377963 PMC3917288

[B12] Herold F. Wiegel P. Scholkmann F. Thiers A. Hamacher D. Schega L. (2017). Functional near-infrared spectroscopy in movement science: a systematic review on cortical activity in postural and walking tasks. *Neurophotonics* 4:041403. 10.1117/1.NPh.4.4.041403 28924563 PMC5538329

[B13] Herzog M. M. Kerr Z. Y. Marshall S. W. Wikstrom E. A. (2019). Epidemiology of ankle sprains and chronic ankle instability. *J. Athl. Train*. 54 603–610. 10.4085/1062-6050-447-17 31135209 PMC6602402

[B14] Hocke L. M. Oni I. K. Duszynski C. C. Corrigan A. V. Frederick B. D. Dunn J. F. (2018). Automated processing of fNIRS data-A visual guide to the pitfalls and consequences. *Algorithms* 11:67. 10.3390/a11050067 30906511 PMC6428450

[B15] Hoshi Y. (2003). Functional near-infrared optical imaging: utility and limitations in human brain mapping. *Psychophysiology* 40 511–520. 10.1111/1469-8986.00053 14570159

[B16] Kim K. M. Kim J. S. Cruz-Díaz D. Ryu S. Kang M. Taube W. (2019). Changes in spinal and corticospinal excitability in patients with chronic ankle instability: a systematic review with meta-analysis. *J. Clin. Med*. 8:1037. 10.3390/jcm8071037 31315231 PMC6678466

[B17] Kohli S. Fitzgibbon-Collins L. K. Luan S. Durand N. Brunton L. Fleet J. (2025). Exploring the relationship between prefrontal cortex activation, standing balance, and fatigue in people post-stroke: a fNIRS study. *NeuroRehabilitation* 57:10538135251341124. 10.1177/10538135251341124 40371461 PMC12657648

[B18] Kumar V. Shivakumar V. Chhabra H. Bose A. Venkatasubramanian G. Gangadhar B. N. (2017). Functional near infra-red spectroscopy (fNIRS) in schizophrenia: a review. *Asian J. Psychiatr*. 27 18–31. 10.1016/j.ajp.2017.02.009 28558892

[B19] Lee Y. Curuk E. Aruin A. S. (2021). Effect of light finger touch, a cognitive task, and vision on standing balance in stroke. *J. Mot. Behav*. 53 157–165. 10.1080/00222895.2020.1742082 32281912

[B20] Lehmann T. Büchel D. Mouton C. Gokeler A. Seil R. Baumeister J. (2021). Functional Cortical Connectivity Related to Postural Control in Patients Six Weeks After Anterior Cruciate Ligament Reconstruction. *Front Hum Neurosci*. 15:655116. 10.3389/fnhum.2021.655116 34335206 PMC8321596

[B21] Li Q. Feng J. Guo J. Wang Z. Li P. Liu H. (2020). Effects of the multisensory rehabilitation product for home-based hand training after stroke on cortical activation by using NIRS methods. *Neurosci. Lett*. 717:134682. 10.1016/j.neulet.2019.134682 31837442

[B22] Li Y. Ko J. Walker M. A. Brown C. N. Schmidt J. D. Kim S. H. (2018). Does chronic ankle instability influence lower extremity muscle activation of females during landing? *J. Electromyogr. Kinesiol*. 38 81–87. 10.1016/j.jelekin.2017.11.009 29175719

[B23] Lin T. T. Cheng L. Y. Chen C. C. Pan W. R. Tan Y. K. Chen S. F. (2024). Age-related influence on static and dynamic balance abilities: an inertial measurement unit-based evaluation. *Sensors* 24:7078. 10.3390/s24217078 39517975 PMC11548656

[B24] Liu N. Yang C. Song Q. Yang F. Chen Y. (2024). Patients with chronic ankle instability exhibit increased sensorimotor cortex activation and correlation with poorer lateral balance control ability during single-leg stance: a FNIRS study. *Front. Hum. Neurosci*. 18:1366443. 10.3389/fnhum.2024.1366443 38736530 PMC11082417

[B25] Ma T. Xu X. Li M. Li Y. Wang Y. Li Q. (2023). Cortical activation during single-legged stance in patients with chronic ankle instability. *J. Athl Train*. 58 927–933. 10.4085/1062-6050-0363.22 36827609 PMC10784888

[B26] Maricot A. Dick E. Walravens A. Pluym B. Lathouwers E. De Pauw K. (2023). Brain neuroplasticity related to lateral ankle ligamentous injuries: a systematic review. *Sports Med*. 53 1423–1443. 10.1007/s40279-023-01834-z 37155129

[B27] Menant J. C. Maidan I. Alcock L. Al-Yahya E. Cerasa A. Clark D. J. (2020). A consensus guide to using functional near-infrared spectroscopy in posture and gait research. *Gait Posture* 82 254–265. 10.1016/j.gaitpost.2020.09.012 32987345

[B28] Mohebbi A. Amiri P. Kearney R. E. (2022). Identification of human balance control responses to visual inputs using virtual reality. *J. Neurophysiol*. 127 1159–1170. 10.1152/jn.00283.2021 35353629

[B29] Nanbancha A. Tretriluxana J. Limroongreungrat W. Sinsurin K. (2019). Decreased supraspinal control and neuromuscular function controlling the ankle joint in athletes with chronic ankle instability. *Eur. J. Appl. Physiol*. 119 2041–2052. 10.1007/s00421-019-04191-w 31321512

[B30] Omaña H. Bezaire K. Brady K. Davies J. Louwagie N. Power S. (2021). Functional reach test, single-leg stance test, and tinetti performance-oriented mobility assessment for the prediction of falls in older adults: a systematic review. *Phys. Ther*. 101:zab173. 10.1093/ptj/pzab173 34244801

[B31] Onofrei R. R. Amaricai E. (2022). Postural balance in relation with vision and physical activity in healthy young adults. *Int. J. Environ. Res. Public Health* 19:5021. 10.3390/ijerph19095021 35564412 PMC9105214

[B32] Onuma R. Hoshi F. Tozawa R. Soutome Y. Sakai T. Jinno T. (2023). Reliability and validity of quantitative evaluation of anticipatory postural adjustments using smartphones. *J. Phys. Ther. Sci*. 35 553–558. 10.1589/jpts.35.553 37405178 PMC10315206

[B33] Perrey S. (2008). Non-invasive NIR spectroscopy of human brain function during exercise. *Methods* 45 289–299. 10.1016/j.ymeth.2008.04.005 18539160

[B34] Petraglia F. Scarcella L. Pedrazzi G. Brancato L. Puers R. Costantino C. (2019). Inertial sensors versus standard systems in gait analysis: a systematic review and meta-analysis. *Eur. J. Phys. Rehabil. Med*. 55 265–280. 10.23736/S1973-9087.18.05306-6 30311493

[B35] Piper S. K. Krueger A. Koch S. P. Mehnert J. Habermehl C. Steinbrink J. (2014). A wearable multi-channel fNIRS system for brain imaging in freely moving subjects. *Neuroimage* 85(Pt 1), 64–71. 10.1016/j.neuroimage.2013.06.062 23810973 PMC3859838

[B36] Richer N. Peterson S. M. Ferris D. P. (2024). Vision is not required to elicit balance improvements from beam walking practice. *Motor Control* 28 480–492. 10.1123/mc.2023-0145 39159924 PMC11849333

[B37] Riemann B. L. Lephart S. M. (2002). The sensorimotor system, part I: the physiologic basis of functional joint stability. *J. Athl. Train.* 37 71–79. 16558670 PMC164311

[B38] Ruddy K. L. Leemans A. Woolley D. G. Wenderoth N. Carson R. G. (2017). Structural and functional cortical connectivity mediating cross education of motor function. *J. Neurosci*. 37 2555–2564. 10.1523/JNEUROSCI.2536-16.2017 28154150 PMC5354316

[B39] Sakai J. (2022). Functional near-infrared spectroscopy reveals brain activity on the move. *Proc. Natl. Acad. Sci. U. S. A*. 119:e2208729119. 10.1073/pnas.2208729119 35709323 PMC9231602

[B40] Stuart S. Vitorio R. Morris R. Martini D. N. Fino P. C. Mancini M. (2018). Cortical activity during walking and balance tasks in older adults and in people with Parkinson’s disease: a structured review. *Maturitas* 113 53–72. 10.1016/j.maturitas.2018.04.011 29903649 PMC6448561

[B41] Suzen E. Tombak K. Simsek B. Colak O. H. Ozen S. (2025). Evaluation of prefrontal cortex activation and static balance mechanisms in adolescent idiopathic scoliosis using fNIRS. *Medicina* 61:667. 10.3390/medicina61040667 40282959 PMC12028968

[B42] Tachtsidis I. Scholkmann F. (2016). False positives and false negatives in functional near-infrared spectroscopy: issues, challenges, and the way forward. *Neurophotonics* 3:031405. 10.1117/1.NPh.3.3.031405 27054143 PMC4791590

[B43] Tomomitsu M. S. Alonso A. C. Morimoto E. Bobbio T. G. Greve J. M. (2013). Static and dynamic postural control in low-vision and normal-vision adults. *Clinics* 68 517–521. 10.6061/clinics/2013(04)13 23778351 PMC3634964

[B44] Tzourio-Mazoyer N. Landeau B. Papathanassiou D. Crivello F. Etard O. Delcroix N. (2002). Automated anatomical labeling of activations in SPM using a macroscopic anatomical parcellation of the MNI MRI single-subject brain. *Neuroimage* 15 273–289. 10.1006/nimg.2001.0978 11771995

[B45] Xie H. M. Xing Z. T. Chen Z. Y. Zhang X. T. Qiu X. J. Jia Z. S. (2022). Regional brain atrophy in patients with chronic ankle instability: a voxel-based morphometry study. *Front. Neurosci*. 16:984841. 10.3389/fnins.2022.984841 36188473 PMC9519998

[B46] Yücel M. A. Lühmann A. V. Scholkmann F. Gervain J. Dan I. Ayaz H. (2021). Best practices for fNIRS publications. *Neurophotonics* 8:012101. 10.1117/1.NPh.8.1.012101 33442557 PMC7793571

[B47] Zhang H. Duan L. Zhang Y. J. Lu C. M. Liu H. Zhu C. Z. (2011). Test-retest assessment of independent component analysis-derived resting-state functional connectivity based on functional near-infrared spectroscopy. *Neuroimage* 55 607–615. 10.1016/j.neuroimage.2010.12.007 21146616

[B48] Ziabari E. Z. Razi M. Haghpanahi M. Lubberts B. Valiollahi B. Khazaee F. (2022a). Does ipsilateral chronic ankle instability alter kinematics of the other joints of the lower extremities: a biomechanical study. *Int. Orthop*. 46 241–248. 10.1007/s00264-021-05139-6 34463806

[B49] Ziabari E. Z. Haghpanahi M. Razi M. Lubberts B. Ashkani-Esfahani S. DiGiovanni C. W. (2022b). The effects of chronic ankle instability on the biomechanics of the uninjured, contralateral ankle during gait. *Orthop. Surg*. 14 2238–2244. 10.1111/os.13307 35852096 PMC9483063

